# Tumor cell migration is inhibited by a novel therapeutic strategy antagonizing the alpha-7 receptor

**DOI:** 10.18632/oncotarget.14545

**Published:** 2017-01-06

**Authors:** Chris Pepper, Henry Tu, Paul Morrill, Sara Garcia-Rates, Chris Fegan, Susan Greenfield

**Affiliations:** ^1^ Division of Cancer and Genetics, Cardiff University School of Medicine, Cardiff, CF14 4XN, UK; ^2^ Neuro-Bio Ltd, Culham Science Centre, Abingdon, OX14 3DB, UK

**Keywords:** alpha-7 receptor, acetylcholinesterase peptide, cyclized variant, metastases, cell migration

## Abstract

A 14mer peptide (T14) derived from the C-terminus of acetylcholinesterase (AChE) selectively activates metastatic breast cancer cells via the alpha-7 nicotinic receptor (α7 nAChR). This naturally occurring peptide is also present in brain, is elevated in Alzheimer's disease, and is antagonised by a cyclized variant (NBP-14). Here we investigated the effects of NBP-14 in six different cancer cell lines, primary leukemia B-cells and normal B-cells. All cells tested expressed α7 nAChR, intracellular and extracellular T14. However, NBP-14 showed low toxicity and weak anti-proliferative effects in the majority of the cell lines and was even less toxic in normal B-cells when compared to primary chronic lymphocytic leukemia cells (*P* < 0.001). Given the potential role of T14 peptide in metastasis, we next investigated the effects of NBP-14 on tumor cell migration, where it caused a dose-dependent reduction. The extent of NBP-14 inhibition positively correlated with the migration of the cells (*r*^2^ = 0.45; *P* = 0.06). Furthermore, NBP-14 preferentially inhibited the migration of primary leukemia cells when compared with normal B-cells (*P* = 0.0002); when the normal B-cell data was excluded, this correlation was strengthened (*r*^2^ = 0.80; *P* = 0.006). Importantly, the constitutive α7 nAChR expression positively correlated with intracellular T14 levels (*r*^2^ = 0.91; *P* = 0.0003) and inversely correlated with extracellular T14 levels in the cell culture supernatants (*r*^2^ = −0.79; *P* = 0.034). However, in the presence of NBP-14, α7 nAChR expression was reduced (*P* = 0.04) and the most migratory cells showed the largest reduction in expression. In conclusion, NBP-14-mediated antagonism of the α7 nAChR offers a novel therapeutic strategy with the potential to inhibit tumor cell migration.

## INTRODUCTION

Nicotinic acetylcholine receptors (nAChRs) are ligand-gated ion channels expressed in the cell membrane of a wide range of mammalian cells, including cancer cells [1]. Recent findings suggest that nAChRs not only mediate diverse functions in the brain, such as learning, memory, addiction and general plasticity [2] but may also contribute to the development and progression of a diverse array of cancers [3]. Binding of acetylcholine to the α subunits of nAChRs results in a conformational change in the receptor allowing ions to flow from the extracellular space into the cell [4]. The influx of the cations causes membrane depolarization which in turn triggers the opening of voltage-activated calcium channels, leading to an additional influx of calcium. This promotes a host of diverse responses, including the release of neurotransmitters and angiogenic, neurotrophic and growth factors. In addition, the increased intracellular cation concentrations may trigger the direct stimulation of intracellular signaling cascades that are involved in the regulation of cell proliferation, apoptosis, migration and differentiation [5].

Over the last 45 years [6] a link between cancer cell proliferation and acetycholinesterase (AChE) has become increasingly well established. In addition to its conventional enzymatic role in hydrolyzing acetylcholine, AChE could have an effect in cancer, independent of cholinergic transmission, that involves a variety of potential functions such as cell adhesion, differentiation, regulation of apoptosis and proliferation [7]. One potential clue for these novel actions of AChE is the shift in patterns of oligomerization characterised during metastases, where the typical tetramer of four catalytic subunits disappears in favour of an increased number of dimers and monomers [8]. A possible explanation for this shift could be that the disulphide bond necessary for oligomerization is no longer intact, due to the cleavage of a constituent 14mer peptide (T14) at the C-terminal of AChE [9], rendering it available to act as a signaling molecule.

In a previous study, we showed that T14 had a selective action on the strongly metastatic breast cancer cell line MDA-MB-231 via the α7 nAChR. In contrast, these effects were not evident on the less metastatic cell line MCF-7 [10]. Accordingly, the initial aim of this study was to investigate whether endogenous α7 nAChR and T14 could be detected in a range of cancer cell lines and primary malignant and non-malignant cells. Furthermore, since recent observations have indicated that the action of the AChE-peptide T14 on brain tissue can be blocked by a cyclized variant (NBP-14) [11], we characterized the effects of this cyclized variant in a range of human cancer cell lines as well as primary chronic lymphocytic leukemia cells and normal B-cells.

Here we show for the first time that the α7 nAChR plays a key role in modulating tumor cell migration. Moreover, there was a positive correlation between intracellular T14 and α7 nAChR expression and an inverse correlation between extracellular T14 derived from the culture media and α7 nAChR expression. We further demonstrate that exposure to the α7 nAChR antagonist and cyclized variant of T14, NBP-14, results in reduced tumor cell migration without any significant loss in cell viability. Furthermore, NBP-14 appears to preferentially effect tumor cell migration as normal B-cells were less sensitive to this cyclic peptide. The combination of these characteristics suggests that targeting the α7 nAChR may be a promising therapeutic strategy particularly in cancers with a strong metastatic potential.

## RESULTS

### Intracellular and extracellular T14 expression correlate with α7 nAChR expression

**Figure 1:**
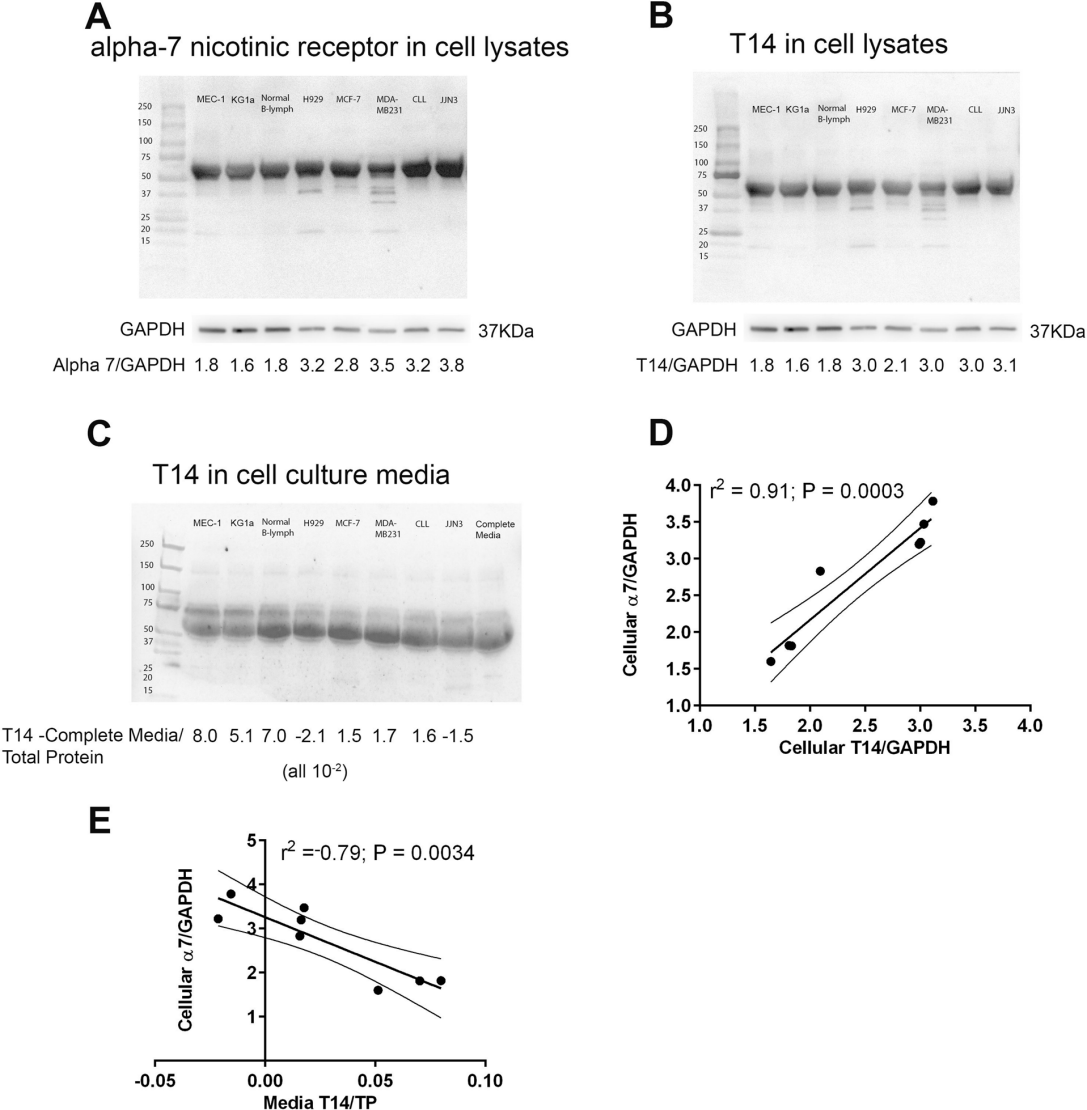
**(A)** α7 nAChR expression was assessed in whole cell lysates (1 × 106 cells) from each cell type using Western blotting. Protein bands were quantified using Image J and the α7 nAChR expression was normalized to GAPDH in order to determine the relative expression of α7 nAChR in the cell lines and primary cells under investigation. **(B)** T14 levels in cell lysates were quantified in the same way and **(C)** the level of T14 present in complete media was subtracted from cell culture media derived from each of cell lines and primary cells, before normalising to total protein using Blot FastStain. **(D)** α7 nAChR positively correlated with intracellular T14 expression (r2 = 0.91, P = 0.0003) and **(E)** negatively correlated with the levels of T14 detected in the culture media (r2 = −0.79, P = 0.0034). Graphs display the lines of best fit together with the 95% confidence intervals.

Our first goal was to determine whether we could detect the T14 peptide in samples collected from each of the cell lines and primary cells. We performed Western blot analysis for T14 and α7 nAChR on cell lysates and aliquots of the cell culture media. The α7 nAChR was not detectable in the cell culture media (data not shown) but was consistently detected in the cell lysates (Figure 1A). α7 nAChR expression levels were normalized to a GAPDH loading control revealing differential expression of α7 nAChR in the various cell lines. In contrast, T14 was detectable in both the cell lysates (Figure 1B) and the cell culture media (Figure 1C). It is worthy of note that T14 was also detected in cell-free media containing 10% fetal calf serum but this could not explain the quantitatively different T14 levels found in the cell lysates and cell culture supernatants. As previously described, T14 was detected as aggregates rather than as a monomeric form [12] thereby explaining the higher than expected molecular weight of the Western blot band. Furthermore, there was a strong positive correlation between intracellular T14 and α7 nAChR expression (Figure 1D; *r*^2^ = 0.91) and an inverse correlation between extracellular T14 derived from the culture media and α7 nAChR expression (Figure 1E; *r*^2^ = −0.79).

### NBP-14 demonstrates low cytotoxic and only modest anti-proliferative effects

**Figure 2:**
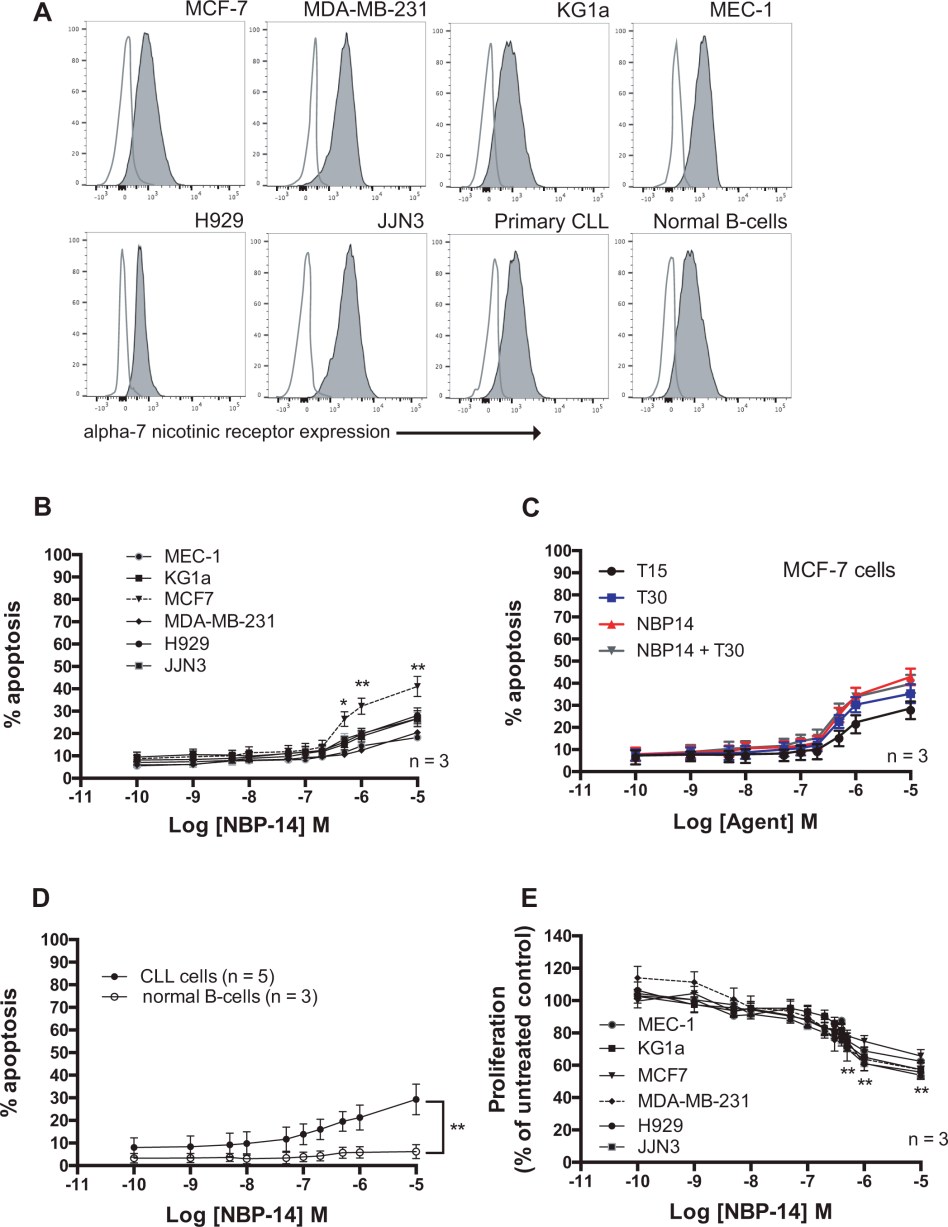
**(A)** Comparison of α7 nAChR expression on the surface of the cancer cell lines and primary cells used in the study. In each case cells not labelled with antibody were analyzed to determine the level of autofluorescence (open histograms). **(B)** Cytotoxic dose-response curves were generated from flow cytometric analysis using Annexin V and propidum iodide labeling of each of the cancer cell lines following exposure to increasing concentrations of NBP-14 for 72 h. **(C)** Comparison of the apoptotic effects of the cyclized peptide (NBP-14), the inert peptide T15, the T30 peptide containing the T14 active peptide amino acid sequence and the combination of NBP-14 and T30 in MCF-7 breast cancer cells. **(D)** The cytotoxic effect of NBP-14 on primary CLL cells (n = 5) and normal B-cells (n = 3). **(E)** NBP-14 induced a dose-dependent decrease in proliferation in all of the cell lines tested. All data are presented as mean (± SD). *P < 0.05 and **P < 0.001.

All of the primary cells and cell lines used in this study were shown to express varying levels of the α7 nAChR (Figure 2A). Treatment with the α7 nAChR antagonist NBP-14 showed modest apoptotic effects in all of the cell lines tested at concentrations > 0.1 μM (Figure 2B). Comparison of the apoptotic effects of NBP-14, T15 and T30 in each of the cell lines are shown in Supplementary Figure 1. Although MCF7 cells showed significantly increased sensitivity to NBP-14, these cells were equally sensitive to the effects of a control peptide (T15) consisting of the inert C-terminal 15 amino acid residues of the neuro-active peptide (T30) suggesting that these effects were non-specific (Figure 2C). Furthermore, NBP-14 was essentially non-toxic in normal B-cells at the concentrations tested and was significantly more toxic in malignant B-cells derived from chronic lymphocytic leukemia (CLL) patients (Figure 2D; *P* < 0.001). In terms of anti-proliferative activity, NBP-14 only showed evidence of cytostatic effects at concentrations of > 0.1 μM (Figure 2E). Comparison of the anti-proliferative effects of NBP-14, T15 and T30 in each of the cell lines are shown in Supplementary Figure 2.

### NBP-14 preferentially inhibits the migration of primary cancer cells

**Figure 3:**
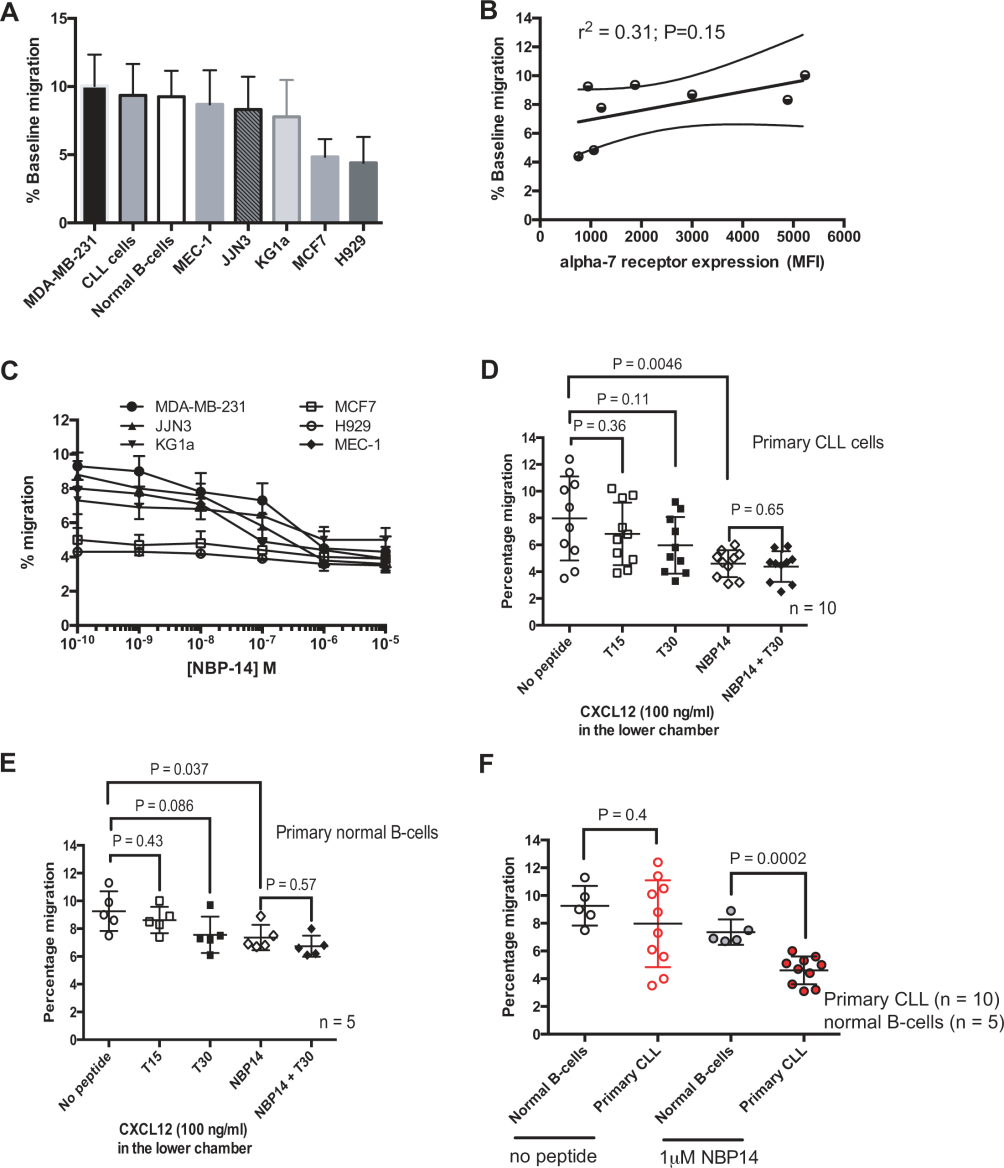
**(A)** Cell migration in transwells was quantified over after 24 h and the mean baseline percentage migration for each of the cell lines and primary cells were arranged in descending order. **(B)** There was a positive correlation (r2 = 0.31) between percentage baseline migration and α7 nAChR expression. **(C)** The inhibitory effect on migration induced by NBP-14 was dose-dependent up to 1 mM for each of the six cancer cell lines tested. Comparison of the anti-migratory responses induced by 1mM of the T15, T30 and NBP-14 peptides in **(D)** primary CLL cells and **(E)** primary normal B-cells. **(F)** Although there was no statistical difference in baseline migration between normal B-cells and CLL B-cells, 1 mM NBP-14 preferentially inhibited the migration of primary CLL cells when compared with normal B-cells.

We next established the migratory potential of all of the primary cells and cell lines employed in this study using transwell assays. There was inherent variation in the propensity of these cells to migrate along a chemokine or serum gradient over a 24 h time period (Figure 3A). Interestingly, there was a positive correlation between α7 nAChR expression, as measured by flow cytometry, and baseline migration of the cell lines and primary cells investigated in this study although this did not reach statistical significance (Figure 3B; *r*^2^ = 0.31). Noting these differences, we examined the effect on migration of the α7 nAChR inhibitory peptide NBP-14 over a range of concentrations. Figure 3C shows dose-response curves for effect of NBP-14 on the migration of the six cancer cell lines. Given that the cell lines (apart from MCF7 cells) showed a significant reduction in migration following exposure to 1 μM NBP-14, we went on to determine the relative effects of 1μM of the inert T15 peptide, the T30 peptide containing the 14 amino acid sequence of T14 and the cyclized NBP-14 peptide in primary CLL cells, normal B-cells (*n* = 5) and each of the cell lines. Samples derived from ten CLL patients showed inherent differences in migration (Figure 3D) but all showed a significant reduction in migration when cultured in the presence of 1 μM NBP-14. In contrast, culture with T15 and T30 had no significant effect. The co-administration of T30 and NBP-14 had no significant effect beyond that achieved with NBP-14 alone. Normal B-cells also showed a significant reduction migration following exposure to NBP-14 (Figure 3E). However, despite manifesting similar levels of basal migration to leukemic CLL cells (*P* = 0.4), normal B-cells were significantly less sensitive to the effects of NBP-14 when compared with malignant B-cells derived from CLL patients (Figure 3F; *P* = 0.0002). It is possible that this may be attributable to the lower levels of α7 nAChR expressed on normal B-cells when compared to primary CLL cells.

### NBP-14 inhibits the migration of diverse cancer cell lines

**Figure 4:**
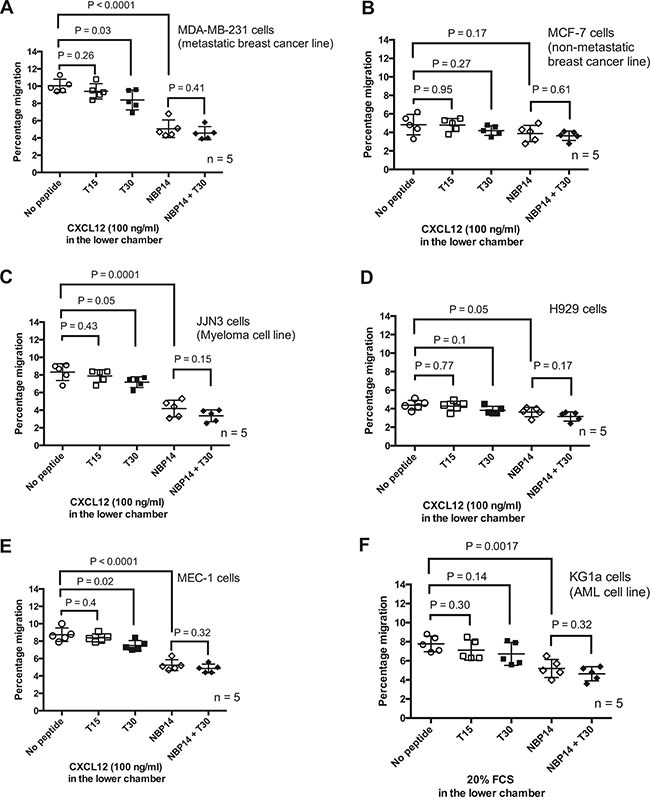
The anti-migratory responses induced by the T15, T30 and NBP-14 peptides were assessed in transwell experiments conducted over 24 h in **(A)** MDA-MB-231 cells **(B)** MCF-7 cells **(C)** JJN3 cells **(D)** H929 cells **(E)** MEC-1 cells and **(F)** KG1a cells. All of the cell lines, apart from MCF7 cells, showed a significant reduction in migration following co-incubation with 1mM NBP-14. The T30 peptide had more variable effects on the cell lines but in every case the inert T15 peptide had no significant impact on the migration of the cells. All data are presented as mean (± SD) of five independent experiments.

Having established that NBP-14 had preferential anti-migratory effects on primary cancer cells, we went on to investigate the effects of NBP-14 on breast cancer, multiple myeloma, acute myeloid leukemia and CLL cell lines (Figure 4A–4F). With the exception of MCF-7 cells, all of the other cell lines showed a significant reduction in migration when treated with 1 μM NBP-14. As was the case with primary CLL cells, the co-administration of T30 and NBP-14 had no additional effect on migration when compared with NBP-14 alone. It is worthy of note that KG1a cells do not express CXCR4, the receptor for the chemokine CXCL12, and so in this case migration was promoted by the addition of 20% FCS into the basolateral chamber of the transwells. NBP-14 was able to inhibit the migration of KG1a cells under these conditions suggesting that the mechanism of inhibition is independent of chemokine receptor expression.

### Basal migration predicts for response to NBP-14

**Figure 5:**
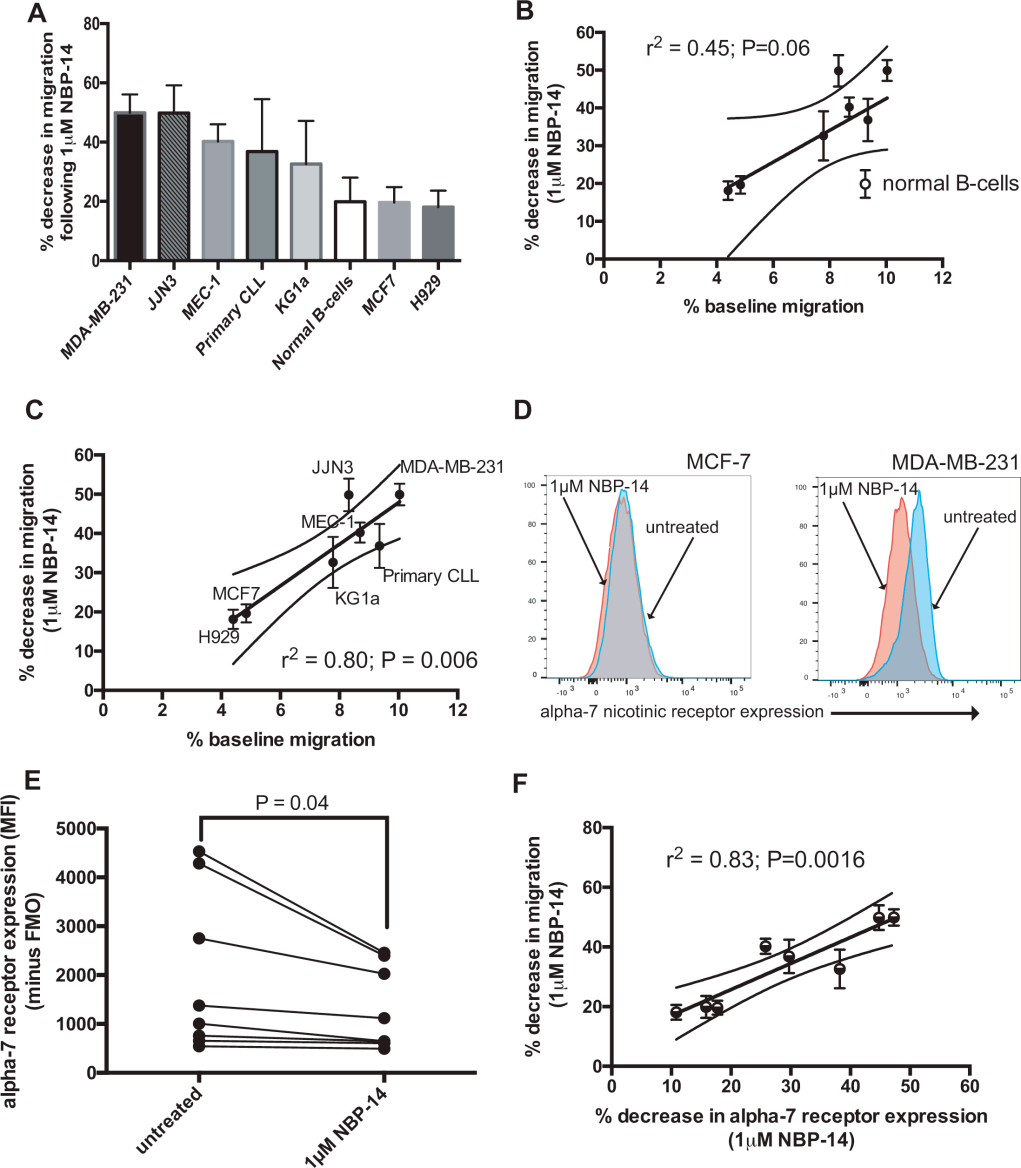
The change in migration induced by 24 h exposure to NBP-14 was calculated for each cell type **(A)** shows the mean percentage decrease in migration induced by 1mM NBP-14 for each of the cell lines and primary cells arranged in descending order. **(B)** We next plotted the change in migration induced by NBP-14 against the percentage baseline migration. These parameters were positively correlated (r2 = 0.45) and **(C)** was strengthened when the normal B-cell data was excluded (r2 = 0.80). **(D)** In parallel experiments, we assessed the effect of exposure to 1 mM NBP-14 on the surface expression of the α7 nAChR on MCF7 and MDA-MB-231 cells. NBP-14 caused a reduction in α7 nAChR expression on both cell lines; this was most apparent on MDA-MB-231 cells, which manifest higher constitutive expression of the receptor. **(E)** Analysis of the other cell lines and primary cells confirmed that α7 nAChR expression was significantly reduced following exposure to 1 μM NBP-14 for 72 h. **(F)** Furthermore, the decrease in migration induced by NBP-14 strongly correlated with the decrease in α7 nAChR expression (r2 = 0.83).

Having established that the different cell lines and primary cells had inherent differences in basal migration, we examined whether this was predictive of response to NBP-14. Figure 5A shows the cell lines ranked according to the percentage decrease in migration induced by 1 μM NBP-14. There was a clear correlation between basal migration and the percentage decrease in migration induced by NBP-14 (Figure 5B; *r*^2^ = 0.45 *P* = 0.06). Furthermore, when the normal B-cells were excluded from the analysis the correlation was even stronger suggesting that tumor cells may be more reliant on T14 signaling in order to migrate (Figure 5C; *r*^2^ = 0.80 *P* = 0.006). Given that we had already established that there was variation in α7 nAChR expression between the cells under investigation, we set out to determine the effect of NBP-14 on α7 nAChR expression. Figure 5D shows that MDA-MB-231 cells showed a marked reduction in α7 nAChR expression following treatment with NBP-14. In contrast, MCF-7 cells showed only a small reduction in α7 nAChR expression under the same conditions. When considering all of the cell types together, there was a significant reduction in α7 nAChR expression following exposure to NBP-14 (Figure 5E; *P* = 0.04). Furthermore, there was a strong positive correlation between the NBP-14-mediated decrease in migration and the percentage decrease in α7 nAChR expression (Figure 5F; *r*^2^ = 0.83, *P* = 0.0016). Although we only analyzed α7 nAChR expression in a small cohort of primary CLL patients (*n* = 6), there was heterogeneity in basal α7 nAChR expression and also the degree of α7 nAChR inhibition induced by NBP-14 (Supplementary Figure 3).

## DISCUSSION

Cancer recurrence at secondary locations, often years after effective treatment of the primary tumor, accounts for most of the mortality associated with solid tumors [13]. Furthermore, the ability of leukemia cells to infiltrate tissues and take advantage of pro-survival microenvironments likely contributes to their chemoresistance. Therefore, understanding the cellular processes that promote metastasis and clinical relapse is of critical importance. In this regard, the identification of novel therapeutic targets is of particular interest because they are less likely to be subject to the common drug resistance mechanisms encountered following treatment with chemotherapy or immunotherapy [14].

AChE has been implicated in tumorogenesis for almost 50 years [6], in many cases in a capacity not linked to its conventional enzymatic role in cholinergic transmission [7]. Of particular interest is the observation that in metastatic mode, there is a shift from a high level of tetramers, to a higher proportion of dimers and monomers [8]. One interpretation of this change in oligomerization pattern is that during metastasis there is an enhanced availability of free C-terminal peptide resulting in significantly increased levels of endogenous T14. A role for T14 in metastases was previously inferred by the effects of exogenous T14 on the highly metastatic breast cancer cell line MDA-MB-231 [10]. Recently a cyclized peptide, NBP-14, has been shown to antagonize the effects of T14 in brain tissue [15]. Using this new agent, we set out to assess whether α7 nAChR antagonism could provide a novel therapeutic target in human cancers. In particular, we wanted to establish whether this peptide could impact upon the migratory capacity of a range of tumor cell lines and primary CLL cells. We compared our findings with those obtained using normal B-cells.

As a first step, we confirmed that all of the cells under evaluation in this study expressed the α7 nAChR. We noted variation in receptor expression but this was not associated with the modest cytotoxic or anti-proliferative effects induced by NBP-14. It was of considerable interest that we were able to detect the active T14 peptide in both cellular extracts and the media that they were cultured in. Indeed, we were able to demonstrate a strong positive correlation between the intracellular levels of T14 and the expression of the α7 nAChR (*r*^2^ = 0.91). Furthermore, there was an inverse correlation between T14 detected in the culture media and expression of the α7 nAChR (*r*^2^ = −0.79).

Despite its lack of potency as a cytotoxic/cytostatic agent, NBP-14 was able to preferentially inhibit the migration of tumor cells when compared to normal B-cells. Moreover, the exogenous, linear peptide T30, when applied alone or in combination with NBP-14 had no significant effect on migration when compared with NBP-14 alone in any of the cell lines and primary cells evaluated. This could be due to a ceiling effect of endogenous T14 preventing any further effects of exogenous T30. This concept was supported by the observation that the most migratory cells manifested the highest levels of α7 nAChR, the lowest levels of extracellular T14 but the highest levels of intracellular T14. This implies that these cells are capable of scavenging T14 from their microenvironment resulting in optimal intracellular signaling. Exposure to the cyclized variant, NBP-14, caused a significant reduction in surface expression of α7 nAChR presenting a plausible mechanism for blocking the actions of endogenous T14.

Primary CLL cells showed baseline heterogeneity in both their α7 nAChR expression and their migratory capacity. However, NBP-14 was able to significantly reduce migration in all of the primary tumor samples tested. It is of particular interest that although normal B-cells showed a similar level of baseline migration to leukemic B-cells, they were significantly less sensitive to the anti-migratory effects of NBP-14 (*P* = 0.0002). This implies normal B-cells are not as dependent on the effects of α7 nAChR signaling as their malignant counter-parts presenting the potential for a positive therapeutic index when inhibiting this target. A rationale for these observed differential effects is that normal B-cells expressed less α7 nAChR when compared with leukemic B-cells. This concept was further supported by the finding that there was a strong positive correlation between the reduction in α7 nAChR expression and the reduction in migration induced by NBP-14 in all of the cell lines and primary cells evaluated.

In conclusion, the findings of this study support the concept that antagonizing α7 nAChR signaling in human cancers represents a promising therapeutic strategy. Although the precise downstream consequences of α7 nAChR signaling remain unresolved, it is clear that the molecular targets relate to migration rather than proliferation and survival. This raises the possibility of combining α7 nAChR antagonists, such as the novel agent NBP-14 described here, with other anti-cancer agents both in the frontline and relapsed/refractory settings.

## MATERIALS AND METHODS

### Culture conditions for cancer cell lines, primary CLL cells and normal B-cells

Primary chronic lymphocytic leukemia (CLL) cells were obtained from patients attending outpatients’ clinics at the University Hospital of Wales with informed consent in accordance with the ethical approval granted by South East Wales Research Ethics Committee (02/4806). Normal B-cells were obtained from healthy volunteers again with informed consent. All of the cell lines were purchased from DSMZ and were used for these experiments within 6 months of purchase. In each case multiplex PCR of minisatellite markers revealed a unique DNA profile and were shown to be mycoplasma-free. All of the cell lines, the primary CLL cells and normal B-cells were maintained in RPMI medium supplemented with 100 units/ml penicillin, 100 μg/ml streptomycin and 10% fetal calf serum. In addition, 100 μM of acetylcholine (Sigma) was added to the culture media to ensure that the availability of acetylcholine was not a limiting factor in these experiments. Cells were treated with a range of concentrations of peptide (0–10 μM); all peptides (T15, T30 and NBP-14) were all synthesized by Genosphere Biotechnologies.

### Purification of CLL and normal B-cells

Freshly isolated peripheral blood B-cells derived from CLL patients and healthy volunteers were isolated from whole blood by positive selection using CD19 microbeads on an AutoMACS Pro separator (Miltenyi Biotec.). Cell purity was subsequently checked using an Accuri C6 flow cytometer (BD Biosciences) after staining an aliquot of cells with a fluorescein isothiocyanate-labeled CD20 antibody (Biolegend). In every case > 94% of the cells analyzed were expressing CD20.

### Measurement of *in vitro* apoptosis

Aliquots of each cell type (1 × 10^6^ cells) were cultured for 72 h, harvested by centrifugation (300 g for 5 mins) and then resuspended in 195 μL of calcium-rich buffer. Subsequently, 5 μL of Annexin V (eBiosciences) was added to the cell suspension and cells were incubated in the dark for 10 mins prior to washing. Cells were finally resuspended in 190 μL of calcium-rich buffer together with 10 μL of propidium iodide. Apoptosis was assessed by dual-color immunofluorescent flow cytometry using an Accuri C6 flow cytometer and data were analyzed using CFlow software (BD Biosciences).

### Measurement of *in vitro* proliferation

Cultured cells were harvested by centrifugation and were then counted using a Vi-Cell XR cell viability counter (Beckman Coulter). The number of viable cells in each culture was then expressed as a percentage of the viable cells in the control cultures (no peptide).

### Western blotting

Whole cell pellets (1 × 10^6^ cells) from eight cancer cell lines (MEC-1, KG1a, JJN3, H929, MCF-7, MDA-MB-231, primary CLL cells and normal B-cells) were solubilized in 1 x Lysis Buffer (20 mM Tris Base, 137 mM NaCl, 1% Tween-20, 2 mM EDTA) containing protease inhibitor cocktails (Phosphatase 1:1, PMSF 1:1, aprotinin 1:1) with a 17% v/v ratio. Subsequently, the mixture was triturated using a Polytron for 10 seconds and shaken at 4°C for 2 h. Then, the samples were centrifuged at 13,000 g for 30 minutes at 4°C and the supernatants were taken and stored at −80°C. Protein concentrations were determined in the cell lysate samples above and their corresponding culture media samples using the Pierce^™^ 660 nm Protein Assay (Thermo Scientific, 22660). Subsequently, Western blot analysis was conducted on the samples using the previous established method [15]. The primary antibodies used were anti-T14 antibody (1:1000) [15], GAPDH (abcam, Ab181602, 1:1000) and anti- α7 nAChR (abcam, Ab10096, 1:1000). The secondary antibody used was goat anti-rabbit antibody conjugated to horseradish peroxidase (Abcam, ab6721, 1:5000). Protein bands derived from the cell lysates were quantified using Image J, measuring total optical intensity, and were subsequently normalized to the GAPDH loading control. In the case of the culture supernatants, T14 and α7 nAChR expression were normalized to total protein levels using Blot FastStain [16] to control for loading error.

### Flow cytometric analysis of Alpha-7 nicotinic receptor expression

Aliquots of 1 × 10^6^ cells from each cell line or primary cell sample were harvested by centrifugation, resuspended in 100 μL staining buffer (PBS + 5% FCS) and then labeled with 10 μL anti-α7 nAChR antibody directly conjugated with phycoerythrin (Santa Cruz Biotech., Sc-58607-PE). Cells were incubated with primary antibodies at 4°C for 20 min in the dark to prevent photobleaching. The cell suspension was then washed in 1 mL of staining buffer and prior to resuspension in 200 μL of 1% paraformaldehyde solution. At least 10,000 cells were analyzed using an Accuri C6 flow cytometer (Becton Dickinson) and data were expressed as mean fluorescence intensity (MFI) values. In order to control for autofluorescence, the α7 nAChR MFI values were corrected by subtracting the FL2 MFI values derived from the analysis of unlabeled cells.

### Drugs and reagent

Acetylthiocholine chloride was provided by Sigma-Aldrich, A5626. T30, T15 and NBP14 were synthesized by Genosphere Biotechnologies (France).

### Migration assays

*In vitro* migration assays were performed by using 6.0 μm pore size transwell migration plates (Costar, Corning). Aliquots (1 × 10^6^ cells) of each cell type in 500 μl of supplemented RPMI media were mixed with the appropriate concentration of peptide and were then added to the upper chamber of the transwell insert. 100 ng/ml of CXCL12 was added to the baso-lateral chamber for all the cell types tested apart from KG1a cells. KG1a cells do not express CXCR4 and so are unresponsive to CXCL12. Instead, media containing 20% fetal calf serum was added to the baso-lateral chamber in these experiments. The plates were incubated for 24h at 37°C in 5% CO_2_ in the presence of the peptides (T15, T30, NBP-14 and the combination of T30+NBP−14) at concentrations between 0.1 nM and 10 μM. In addition, control cultures were carried out to which no peptide was added. Cells were subsequently harvested by centrifugation and were analyzed by flow cytometry using an Accuri C6 flow cytometer (BD). None of the conditions tested induced significant cell death in the cultures. Migration of CLL cells was determined by counting cells that migrated to the lower (baso-lateral) chamber of the transwell plate and then expressed as a percentage of the total number of cells initially added to the upper (apical) chamber.

### Statistical analysis

The data obtained in these experiments were evaluated using the Wilcoxon signed rank test and correlation coefficients were calculated from least squares linear regression plots. LD_50_ values were calculated from line of best-fit analysis of the dose-response curves. All statistical analyses were performed using Graphpad Prism 6.0 software (Graphpad Software Inc., San Diego, CA).

## SUPPLEMENTARY MATERIALS FIGURES AND TABLES



## References

[1] Wessler I, Kirkpatrick CJ (2009). Acetylcholine beyond neurons: The non-neuronal cholinergic system in humans. Br J of Pharmacol.

[2] Nees F (2015). The nicotinic cholinergic system function in the human brain. Neuropharmacol.

[3] Schuller HM (2009). Is cancer triggered by altered signalling of nicotinic acetylcholine receptors?. Nat Rev Cancer.

[4] Le Novere N, Changeux JP (1995). Molecular evolution of the nicotinic acetylcholine receptor: An example of multigene family in excitable cells. J Mol Evol.

[5] Roderick HL, Cook SJ (2008). Ca2+ signalling checkpoints in cancer: Remodelling ca2+ for cancer cell proliferation and survival. Nat Rev Cancer.

[6] Blume A, Gilbert F, Wilson S, Farber J, Rosenberg R, Nirenberg M (1970). Regulation of acetylcholinesterase in neuroblastoma cells. Proc Natl Acad Sci USA.

[7] Xi HJ, Wu RP, Liu JJ, Zhang LJ, Li ZS (2015). Role of acetylcholinesterase in lung cancer Thorac. Cancer.

[8] Vidal CJ (2005). Expression of cholinesterases in brain and non-brain tumours. Chem Bio Interact.

[9] Greenfield S (2013). Discovering and targeting the basic mechanism of neurodegeneration: The role of peptides from the c-terminus of acetylcholinesterase. Chem Bio Interact.

[10] Onganer PU, Djamgoz MBA, Whyte K, Greenfield SA (2006). An acetylcholinesterase-derived peptide inhibits endocytic membrane activity in a human metastatic breast cancer cell line Biochim. Biophys Acta – Gen Subjects.

[11] Badin AS, Morrill P, Devonshire IM, Greenfield SA (2016). (II) physiological profiling of an endogenous peptide in the basal forebrain: Age-related bioactivity and blockade with a novel modulator. Neuropharmacol.

[12] Cottingham M, Hollinshead Vaux D (2002). Amyloid fibril formation by a synthetic peptide from a region of human acetylcholinesterase that is homologous to the Alzheimer's amyloid-beta peptide. Biochem.

[13] Coghlin C, Murray GI (2010). Current and emerging concepts in tumour metastasis. J Pathol.

[14] Kachalaki S, Ebrahimi M, L Mohamed Khosroshahi, Mohammadinejad S, Baradaran B (2016). Cancer chemoresistance; biochemical and molecular aspects: A brief overview. Eur J Pharm Sci.

[15] Garcia-Ratés S, Morrill P, Tu H, Pottiez G, Badin AS, Tormo-Garcia C, Heffner C, Coen CW, Greenfield SA. I (2016). pharmacological profiling of a novel modulator of the α7 nicotinic receptor: Blockade of a toxic acetylcholinesterase-derived peptide increased in Alzheimer brains. Neuropharmacol.

[16] Collins M, An J, Peller D, Bowser R (2015). Total protein is an effective loading control for cerebrospinal fluid western blots. J Neurosci Methods.

