# Supplementary material for: Tumor cell migration is inhibited by a novel therapeutic strategy antagonizing the alpha-7 receptor
Source: Oncotarget. 2017 Jan 6;8(7):11414–24. doi: 10.18632/oncotarget.14545 (PMC5355275; doi:10.18632/oncotarget.14545)
Supplement: Supplementary file 1 [file oncotarget-08-11414-s001.pdf]

Tumor cell migration is inhibited by a novel therapeutic strategy antagonizing the alpha-7 receptor

Supplementary Materials

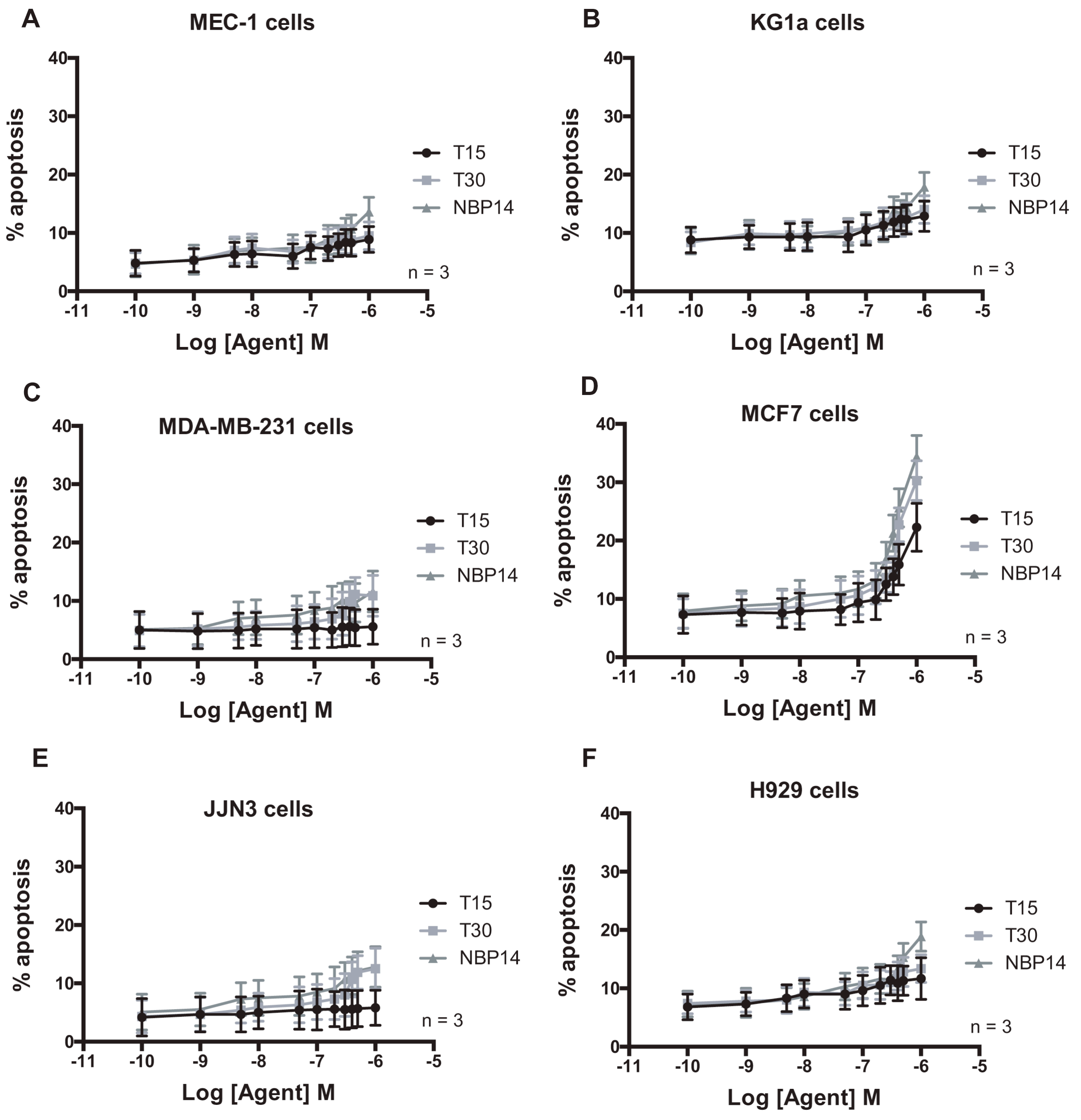

Supplementary Figure 1: The apoptotic effect of NBP-14, T15 and T30 peptides on (**A**) MEC-1 cells, (**B**) KG1a cells, (**C**) MDA-MB-231 cells, (**D**) MCF7 cells, (**E**) JJN3 cells and (**F**) H929 cells. In all cases apoptosis was quantified using Annexin V / propidium iodide labeling using flow cytometry. The results are shown as the mean ± standard deviation of three independent experiments.

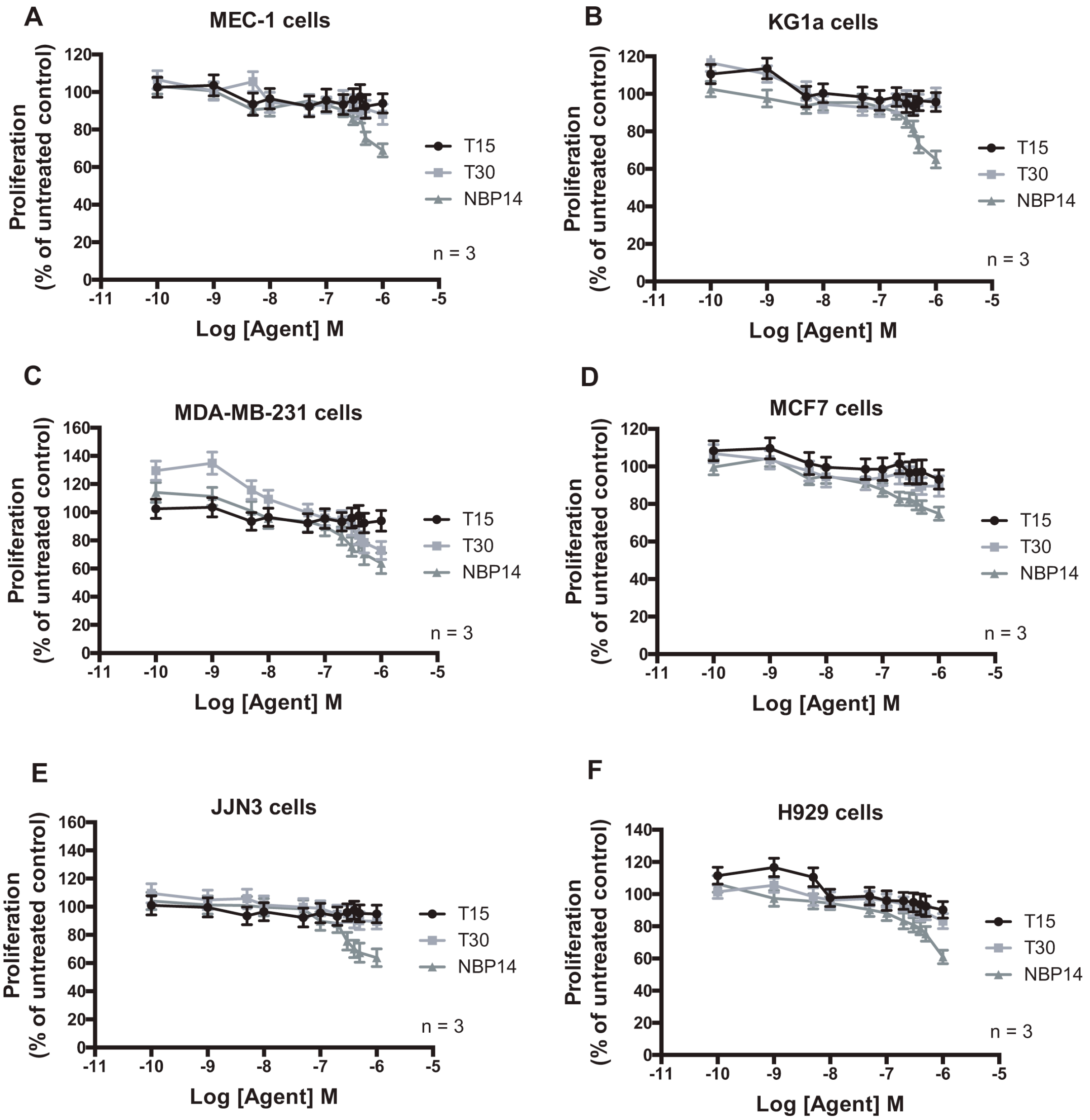

Supplementary Figure 2: The anti-proliferative effect of NBP-14, T15 and T30 peptides on (**A**) MEC-1 cells, (**B**) KG1a cells, (**C**) MDA-MB-231 cells, (**D**) MCF7 cells, (**E**) JJN3 cells and (**F**) H929 cells. Proliferation was measured by monitoring cell number in each culture and expressed as a percentage of the number of viable cells in the control cultures (no peptide).

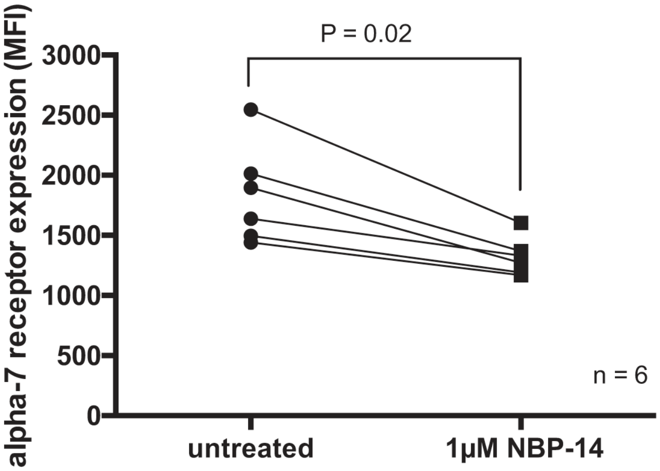

Supplementary Figure 3: Primary CLL samples show inherent variation in α7 nAChR expression. Exposure to 1 μM NBP-14 resulted in a significant reduction in surface expression of the receptor as measured by flow cytometry.
